# Supplementary material for: KPop: accurate and scalable comparative analysis of microbial genomes by sequence embeddings
Source: Genome Biol. 2025 Jun 18;26:170. doi: 10.1186/s13059-025-03585-8 (PMC12175428; doi:10.1186/s13059-025-03585-8)
Supplement: Supplementary file 1 — Additional file 1. KPop-supplementary-figures.pdf. KPop: accurate and scalable comparative analysis of microbial genomes by sequence embeddings - Supplementary figures. [file 13059_2025_3585_MOESM1_ESM.pdf]

KPop: accurate and scalable comparative analysis of microbial genomes by sequence embeddings

Xavier Didelot[1] and Paolo Ribeca[2]

[1] *School of Life Sciences and Department of Statistics, University of Warwick, Coventry, UK*
[2] *Biomathematics and Statistics Scotland, The James Hutton Institute, Edinburgh, UK*
[2] *UK Health Security Agency, London, UK*

April 22, 2025

Supplementary figures

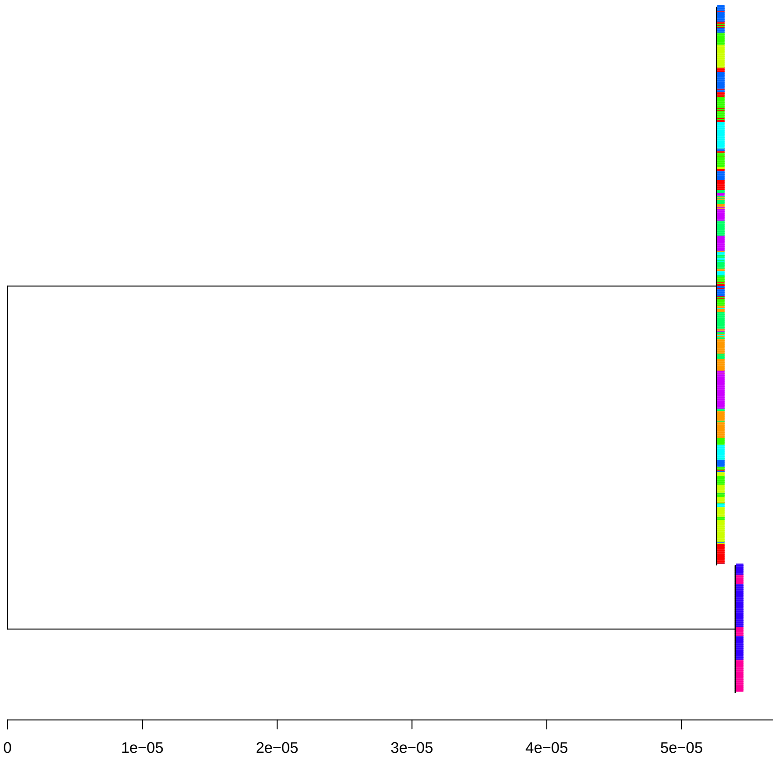

Figure S1. *Tree produced by MashTree on the simulated tuberculosis dataset.* The leaves of the tree are shown using unique colours for each of the ten lineages as in Figure 3.

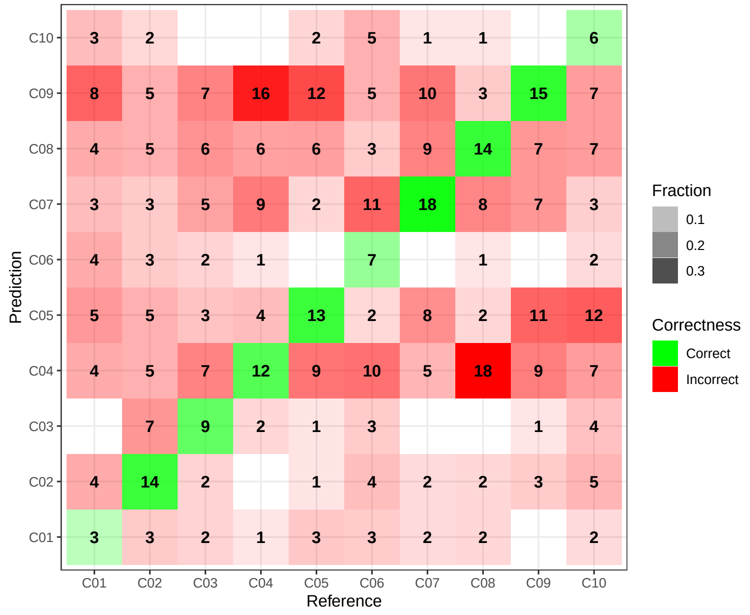

Figure S2. *Confusion matrix for the results of* `sourmash tax` *on the simulated tuberculosis dataset at* $k = 31$. The ten classes in the figure (named C1 to C10) correspond to the ten lineages in Figure 3.

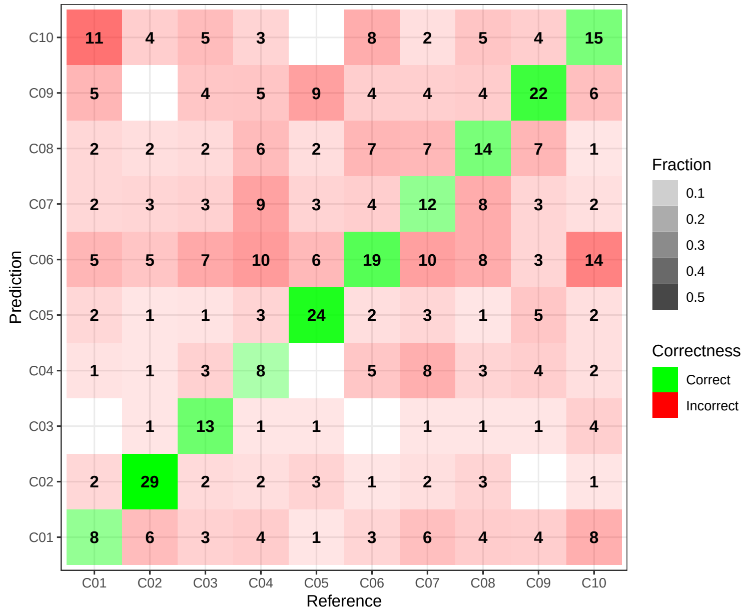

Figure S3. *Confusion matrix for the results of* `sourmash tax` *on the simulated tuberculosis dataset at* $k = 21$. The ten classes in the figure (named C1 to C10) correspond to the ten lineages in Figure 3.

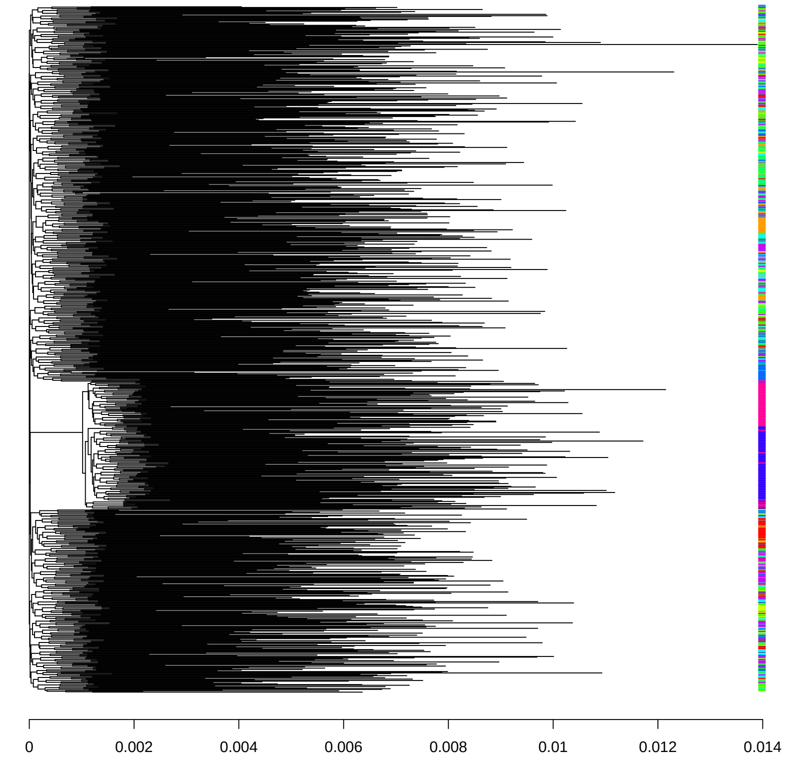

Figure S4. *Neighbour-joining tree based on the distances estimated by* `sourmash` *on the simulated tuberculosis dataset.* The leaves of the tree are shown using unique colours for each of the ten lineages as in Figure 3.

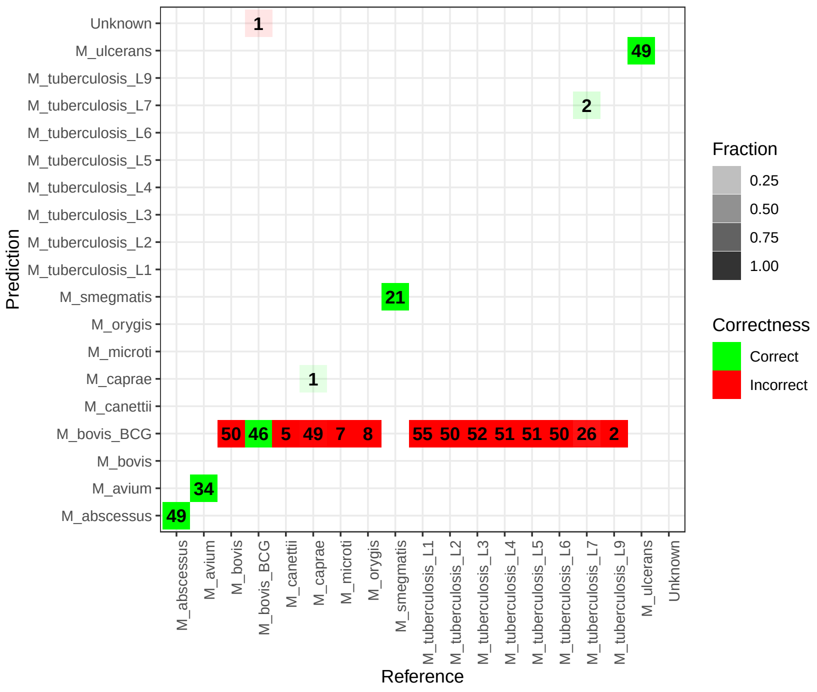

Figure S5. *Confusion matrix for the results of* `sourmash tax` *on the Mycobacterium sequencing dataset at* $k = 31$*.*

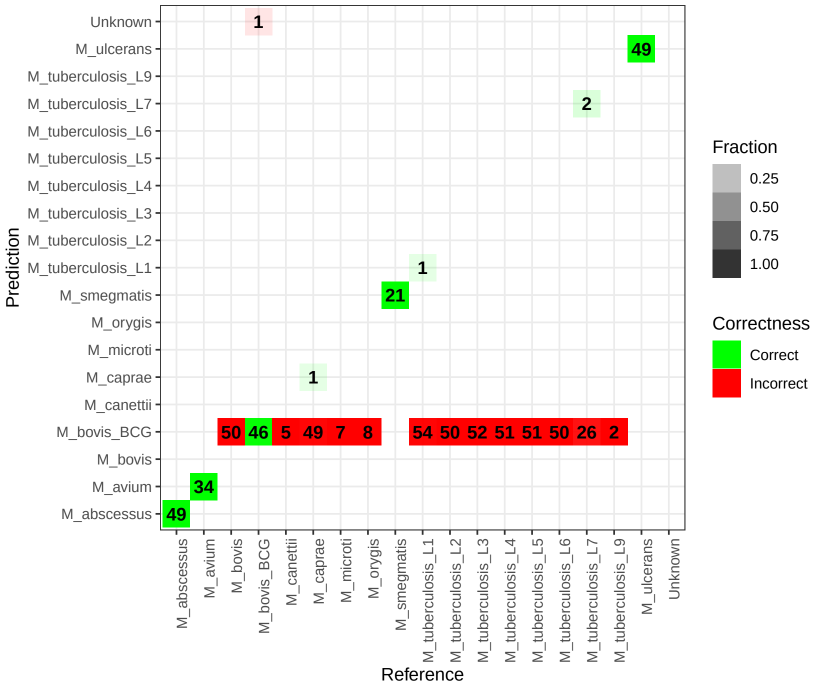

Figure S6. *Confusion matrix for the results of* `sourmash tax` *on the Mycobacterium sequencing dataset at* $k = 21$*.*

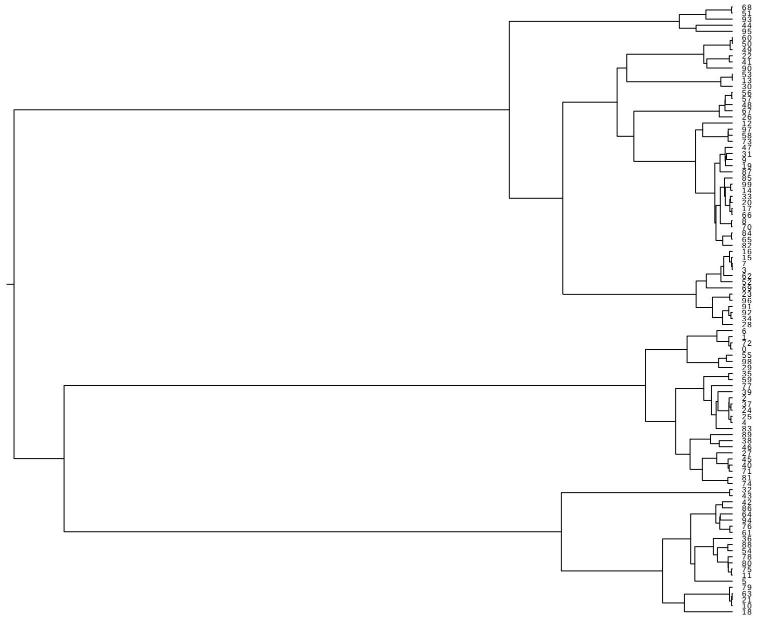

Figure S7. *Clonal genealogy used by SimBac when simulating the dataset of 100 genomes from a recombinant population.*

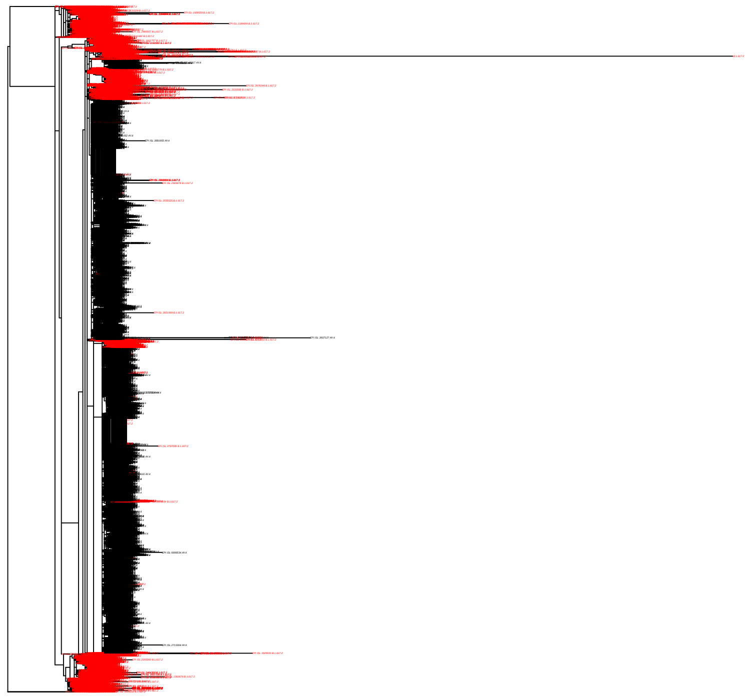

Figure S8. *Tree produced by Usher on the SARS-CoV-2 dataset, showing the genomes from lineage B.1.617.2 in red and the genomes from lineage AY.4 (alias of B.1.617.2.4) in black.*
