# Supplementary material for: KPop: accurate and scalable comparative analysis of microbial genomes by sequence embeddings
Source: Genome Biol. 2025 Jun 18;26:170. doi: 10.1186/s13059-025-03585-8 (PMC12175428; doi:10.1186/s13059-025-03585-8)
Supplement: Supplementary file 2 — Additional file 2. Review history [file 13059_2025_3585_MOESM2_ESM.docx]

**1^st^ round**

**Reviewer 1**

Didelot et al., present a new method 'KPop' that uses Correspondence Analysis (CA) applied to a K-mer frequency spectrum to compare and classify assembled and unassembled microbial genomes. This addresses an important problem in microbial genomics. While the description of the method is compelling, and several insightful examples are provided the results are currently insufficient to support the author's claims. Additional, more extensive examples and comparisons with alternative approaches are required. Moreover, although I managed to install the software, I had some difficulty in getting it to run successfully as described below.

Major Issues:

- Currently, there is no comparison with alternative approaches and thus claims about the superiority of the method are not adequately supported. I think at a minimum, the results of running the other algorithms mentioned including poppunk and mash should be included for both the simulated and real datasets. As this is a K-mer based method, it would also be good to compare the results with fastANI (Jain et al., 2018) as this is a highly popular alternative algorithm. For the SARS-CoV-2 classification example, I think it is necessary to compare with usher as this is the current state of the art (Turakhia et al., 2021).

- The authors argue that KPop is insensitive to recombination (p 4, line 41). However, the simulations and datasets they examine primarily consist of clonally evolving pathogens. These species tend to possess less diverse pangenomes, which reduces the number of unique K-mers. It would be beneficial for the authors to consider larger datasets featuring species that frequently recombine, such as E. coli or K. pneumoniae, in addition to conducting further simulations that incorporate recombination and horizontal gene transfer. The extensive curated dataset of bacterial genomes in Blackwell et al., 2021 (doi.org/10.1371/journal.pbio.3001421) might be a useful test case.

- The method uses Correspondence Analysis to perform dimensionality reduction or the 'twist.' As the authors note (p4, line 6), this polynomial-time algorithm is resource-intensive. Consequently, the authors propose performing optimization on a representative subset. The implications of using only a subset in the optimisation process warrants more careful consideration. A plot illustrating the decrease in performance as fewer genomes are included in the optimization step would help address this concern.

- Approximate timings for running the KPop algorithm are currently included. As one of the major selling points is the scalability of the approach it is important that the timing of alternative algorithms is considered and compared.

- The authors showcase the method's success by conducting several classification tasks. Population structure and biased sampling can significantly affect the accuracy of methods like KPop in classifying genomes. The authors should evaluate the impact of biased sampling more rigorously. They briefly address this in the SARS-CoV-2 simulation, where they identify an error attributed to a small class (p10, line 46). In addition to conducting further simulations, the authors should explain how the presence of classes not included in the training data would impact their approach.

- p14, line 7: The authors state that the package is available on Conda. However, the GitHub repository states that the Conda installation is 'coming soon' and I was unable to find the program in bioconda.

Minor Issues:

- I could install the program using the supplied binaries but was not able to successfully run it. When attempting to run the 'Twist' command:

./KPopTwist -i testDB

I encountered the error

Tools.Argv.parse: Unknown option '' "

- The documentation could be improved by adding a quick start guide that runs the user through building a database and classifying the closest genome without the use of the more complicated parallelisation commands.

- p2, line 13: This appears to suggest k-mer based approaches are insensitive to recombination (including HGT) and should be reworded.

- p2, line 39: I think 'full k-mer spectrum' needs to be defined clearly here. I had to read the methods to understand exactly what was meant by this term.

- p4, line 52: minimiser based methods such as Mash and Poppunk also retain a representation of each sample as a vector in a reduced dimension space. Thus, I think this point needs to be removed.

**Reviewer 2**

Didelot and Ribeca developed a new k-mer-based method, KPop, to efficiently compare microbial genomes. While the authors point out that the method could be developed further to reconstruct phylogeny, in this manuscript the authors show that the method correctly assigns assembled and unassembled genomes to genetic lineages within the same species. The authors tested their method with simulated datasets and some real-life datasets of viruses and bacteria. The key difference between KPop and other k-mer-based methods for comparative genomics is that it is based on full k-mer spectra and that it then transforms the full spectra using a transformation of dimensionality that is specific to the dataset that is being analyzed. The authors claim that this approach combines computational efficiency with higher resolution than that achievable by methods that use reduced k-mer spectra, that is, subsets usually called "sketches" or "signatures".

The manuscript is generally written well. The flow charts help a lot. However, the method is not easy to understand by biologists and, I assume, even by some bioinformaticians. I list some questions below so the authors can improve the explanation of the method. As for the efficiency of the method, the one concern I have is that there is no comparison to other methods. The authors make the reasonable claim that the resolution of KPop is higher than that of Mash because Mash uses reduced k-mer spectra but there are no data to support that claim. Also, the authors do not seem to be aware of the sourmash method, https://sourmash.readthedocs.io/en/latest/ , which is similar to Mash, and can also be used on unassembled sequences. The sourmash tool can use different scaling factors from 1 (no reduction) to 1000 or higher (using only 1 in a thousand k-mer of the k-mer spectrum). Therefore, my main comment is that the authors should not make a claim that their results are better than those of other methods, like Mash and sourmash (although not specifically mentioned), without showing results from a comparison of these methods. The authors say that a direct comparison with Mash is not possible (page 13, line 31-34) but accuracy of classification using Mash or sourmash with KPop should be possible.

Also, what is the actual resolution of KPop? If you had two outbreaks of Escherichia coli or Listeria monocytogenes, could KPop distinguish them from each other? I suggest to use the datasets by Timme et al https://www.ncbi.nlm.nih.gov/pmc/articles/PMC5782805/ and report if KPop can correctly assign a query genome to different outbreaks. At the end, I think, that is one of the main applications envisioned for KPop.

Some more specific questions/comments follow:

In the abstract, the only indication of the high resolution of the method is the word "lineages" implying that KPop can be used to distinguish genomes of closely related strains belonging to the same species. I suggest to point out the advantage of KPop to distinguish closely related strains more specifically.

In the general overview of the workflow, the authors say "KPop can be applied to samples with an arbitrary k-mer composition. What is meant with "arbitrary" here? What would be a "non-arbitrary" k-mer composition? Also, none of the other k-mer-based methods I am aware of requires read alignment and several other methods do not require assembly. Therefore, this is not something specific to KPop but it is a general advantage of k-mer-based methods. Therfore, this should be clarified.

Same as above, k-mer based methods in general are not limited to core genes and generally use all (or a representation of all) genes in a genome.

Figure 2B is not intuitive to understand. The authors should explain the location of the dots on the plot and why that shows that the method worked. Also, the authors talk about a "closest class (page 6, line 48) but no description of what breadth these classes have is provided. In lines 60-62, the authors talk about "closely related". That is a vague term. I suggest to report how many SNPs distinguished classes and how many SNPs distinguished these lineages. This goes back to my overall question of resolution: how different do two classes need to be for KPop to correctly assign genomes to them?

In Figure 6, the color scheme should be explained better. I guess that red represents the higher confidence but that is not clear from the legend.

Choice of k-mer. The authors point out on page 14, lines 51-56, that the choice of k needs to be empirically chosen and be the "smallest value for which the results become stable". I guess that many readers have no idea what that means and would get stuck here before even starting to use KPop. Do the authors provide a detailed documentation with an example dataset to show to users how to choose k for their dataset? Also, it would be useful to report in the various result sections which k was used. That would help users choose k when they want to use Kpop with similar datasets.

Finally, could the author describe, or give an example, of how the actual results look like? For example, having a set of classes and a set of query genomes and running KPop, in what format is the user given the results to which class each query genome belongs? I guess it is a table but it would be useful to have a short paragraph that describes what the output files look like and how to read them as part of the manuscript.

**Reviewer 3**

Didelot and Ribeca present KPop, a k-mer based method for assembly free genome comparisons. The innovation in this approach seems to be a transformation of the k-mer profiles termed "twister" which allows for more efficient comparisons by excluding unimportant k-mers.

Major comments:

The manuscript is written in a style that is often used in computer science conference submissions, which might impede readability for the average Genome Biology reader and in it's current form, the manuscript fails to convince readers that this is not just "another k-mer method".

One of the biggest shortcomings of the manuscript is the complete lack of comparative benchmarking. It is undenyable that other tools exist that can perform the analyses and task that KPop is advertised to do, so the question is: does this tool excel in certain tasks and is it better than other tools. Such benchmarks should include other tools the authors mentioned but also specific adaptations such as sourmash and kmer based tools with specific purpose such as ska + kSNP and Kraken.

How does KPop perform at larger phylogenetic / taxonomic distances? Genus, Family, etc

What happens when the correct "class" is missing? (Also, using "class" in anything about taxonomic assignment is obviously not optimal as Class is also a taxonomic rank)

The results are a mix of methods and results and discussion. Since this is a methods paper, some methods have to be in the results but the authors should carefully check the manuscript to make distinctions clear.

Minor comments:

Regarding the existing evaulation of the method: What is the value of reporting that the second closest class is the correct one? Please explain this within the manuscript

Page 9, Line 42-49: Without showing some examples and investigating them in-depth this statement should be removed. Annotations are always an issue, but this seems very handwavy.

Page 9, Line 50: Could the authors share why sequencing technology or coverage (above a certain minimal threshold that the authors also implement in some form) would influence their or any similar method?

Page 10, Line 60: It would be good if the authors could explain this significantly worse accuracy for SARS-CoV-2

Figures:

There has to be a better visualization of the results. Fig.2bc, Fig5b and Fig6 are all quite unituitive.

**Authors’ response**

We thank the Reviewers for their thorough revision of the manuscript. According to their suggestions, we have now added a number of comparisons with other methods and reworked significant parts of the text in order to make it clearer and to incorporate into it the results of our new tests. We do believe that this revision represents a significant improvement with respect to the previous version; we hope the Editor and the Reviewers will agree and recognise that a significant amount of work has gone into it.

Reviewer #1

Didelot et al., present a new method ’KPop’ that uses Correspondence Analysis (CA) applied to a K-mer frequency spectrum to compare and classify assembled and unassembled microbial genomes. This addresses an important problem in microbial genomics. While the description of the method is compelling, and several insightful examples are provided the results are currently insufficient to support the author’s claims. Additional, more extensive examples and comparisons with alternative approaches are required. Moreover, although I managed to install the software, I had some difficulty in getting it to run successfully as described below.

Major Issues:

• Currently, there is no comparison with alternative approaches and thus claims about the superiority of the method are not adequately supported. I think at a minimum, the results of running the other algorithms mentioned including poppunk and mash should be included for both the simulated and real datasets. As this is a K-mer based method, it would also be good to compare the results with fastANI (Jain et al., 2018) as this is a highly popular alternative algorithm. For the SARS-CoV-2 classification example, I think it is necessary to compare with usher as this is the current state of the art (Turakhia et al., 2021).

We have now added extensive comparisons and partially rewritten the manuscript in order to reflect them. One of the key messages of our paper, and a reason why we had not initially added comparisons, is that our KPop method is able to directly provide an embedding of sequences into a low-dimensional space, while the methods mentioned by the Reviewer only allow for the computation of genomic distances between sequences. I.e., KPop is able to transform sequences into vectors in a low-dimensional space, from which distances can then be computed if needed, while other methods are only able to compute distances. That is an essential feature as it allows us to train classifiers directly from vectors, which is something that other methods cannot do and makes direct comparisons difficult. That is also the main reason why we had originally refrained from comparing KPop with other methods. We have now clarified these concepts in the new Figure 8. We have also added several comparisons with minimiser-based methods (mash, Sourmash) done as explained in the last section of the Methods, i.e., using a K-NN classifier that can be trained from distances alone. Note that we were reluctant to be too critical of existing methods such as mash or Sourmash, because, despite being currently mis-used out of their applicability range, they were never explicitly presented by their authors as able to address the type of fine-scale classification problems that we solve here. Furthermore, we were unable to the best of our skills and available computational power to run some of the other methods proposed by the Reviewer — for example, fastANI requires to perform a computation for every pair of genomes which is very slow and quickly becomes unfeasible. For the SARS-CoV-2 dataset, we do already compare to the Pangolin designation which is state-of-the-art, but it is difficult to interpret discrepancies as discussed in the text. The best comparisons are therefore done on simulated data, where we know what the correct classifications are, and we have added several such benchmarks now.

• The authors argue that KPop is insensitive to recombination (p 4, line 41). However, the simulations and datasets they examine primarily consist of clonally evolving pathogens. These species tend to possess less diverse pangenomes, which reduces the number of unique K-mers. It would be beneficial for the authors to consider larger datasets featuring species that frequently recombine, such as E. coli or K. pneumoniae, in addition to conducting further simulations that incorporate recombination and horizontal gene transfer. The extensive curated dataset of bacterial genomes in Blackwell et al., 2021 (doi.org/10.1371/journal.pbio.3001421) might be a useful test case.

We have now added an analysis of a simulated dataset under recombinant conditions, emulating the evolution of a bacterial species such as Salmonella enterica. Our classification in these conditions was excellent, showing that KPop is indeed insensitive to recombination. The classification obtained using sourmash on the same dataset was far inferior.We also thank the Reviewer for the suggestion to analyse the dataset of Blackwell et al 2021 – we have added a new section to show that we have perfect results at classification of this data at the bacterial species level.

• The method uses Correspondence Analysis to perform dimensionality reduction or the ’twist.’ As the authors note (p4, line 6), this polynomial-time algorithm is resource-intensive. Consequently, the authors propose performing optimization on a representative subset. The implications of using only a subset in the optimisation process warrants more careful consideration. A plot illustrating the decrease in performance as fewer genomes are included in the optimization step would help address this concern.

We have rephrased this sentence and added new text to clarify the meaning. What we meant is that the optimisation of the twister could be performed on a random subset or after pre-clustering the k-mer spectra to obtain a representative subset of the content. In practice, we found that this was not needed for any of the applications in the paper. For any classification problem, we typically know in advance from the training set what the number of classes is – that is usually dictated by the underlying phylogeny – and therefore it is easy to select a representative sample, with the number of classes being typically low. We only wanted to mention that even when the problem implies manipulation of a very large number of sequences, i.e. millions, it would still be possible to devise reasonable strategies that make memory consumption reasonable while at the same time not losing much information.

• Approximate timings for running the KPop algorithm are currently included. As one of the major selling points is the scalability of the approach it is important that the timing of alternative algorithms is considered and compared.

We have now added some timings for other methods.

• The authors showcase the method’s success by conducting several classification tasks. Population structure and biased sampling can significantly affect the accuracy of methods like KPop in classifying genomes. The authors should evaluate the impact of biased sampling more rigorously. They briefly address this in the SARS-CoV-2 simulation, where they identify an error attributed to a small class (p10, line 46). In addition to conducting further simulations, the authors should explain how the presence of classes not included in the training data would impact their approach.

If a class is not present in the training set then it is obviously impossible to classify an object according to it. However, our method might be able to hint to the fact that the sequence belongs to a new class, and that a new class exists, by returning a large distance from the representatives of all classes. However it is not straightforward to define “new” classes in a phylogenetic context as the classification is usually given by the population structure of the pathogen – so we feel like performing tests would go beyond the scope of this paper.

• p14, line 7: The authors state that the package is available on Conda. However, the GitHub repository states that the Conda installation is ’coming soon’ and I was unable to find the program in bioconda.

We have removed this statement and recommend to install from the source code or the binaries available on GitHub instead. In fact we have also started the process of having KPop installable from conda, but this is not ready yet.

Minor Issues:

• I could install the program using the supplied binaries but was not able to successfully run it. When attempting to run the ’Twist’ command:

./KPopTwist -i testDB

I encountered the error

Tools.Argv.parse: Unknown option ’’

As at the moment KPopTwist is a bash script calling KPopTwist , and the error is emitted by the latter, we believe this problem might be due to the Reviewer using a version of env or bash that is not recent enough. The Reviewer is also recommended to add KPopTwist and KPopTwist to their PATH so that the command can be called without pathnames as KPopTwist -i testDB In the meantime we have also have made several changes to the code, so the Reviewer would be encouraged to upgrade to the latest release and try again, as the problem might now have solved itself.

• The documentation could be improved by adding a quick start guide that runs the user through building a database and classifying the closest genome without the use of the more complicated parallelisation commands.

We have now added a quick start guide to the GitHub repository and to the documentation. As suggested by the reviewer, the guide does not use parallelisation commands; together with the new Figure 8 (which details the overall KPop strategy and data formats) and Figure 9 (which details the main possible transformations that can be performed with each command) the quick start guide should hopefully allow users to immediately understand whether their installation has been successful, and represent a simple(r) starting point to implement their own workflows.

• p2, line 13: This appears to suggest k-mer based approaches are insensitive to recombination (including HGT) and should be reworded.

We have removed this sentence which was open to misinterpretation.

• p2, line 39: I think ’full k-mer spectrum’ needs to be defined clearly here. I had to read the methods to understand exactly what was meant by this term.

We have added a sentence to clarify what is meant by full k-mer spectrum.

• p4, line 52: minimiser based methods such as Mash and Poppunk also retain a representation of each sample as a vector in a reduced dimension space. Thus, I think this point needs to be removed.

Although strictly speaking it is true that minimiser-based methods can be seen as representing a sample as a vector in a reduced-dimension space, still the dimension of such space would be very large (of the order of the number of minimisers present in the intersection of all sketches), making the direct use of sketches as vectors impossible from a practical standpoint. Anyway we have rephrased this sentence and also added a detailed comparison with approaches relying on minimiser-based methods in the new Figure 8 and at the end of the Methods section.

Reviewer #2

Didelot and Ribeca developed a new k-mer-based method, KPop, to efficiently compare microbial genomes.

While the authors point out that the method could be developed further to reconstruct phylogeny, in this manuscript the authors show that the method correctly assigns assembled and unassembled genomes to genetic lineages within the same species. The authors tested their method with simulated datasets and some real-life datasets of viruses and bacteria. The key difference between KPop and other k-mer-based methods for comparative genomics is that it is based on full k-mer spectra and that it then transforms the full spectra using a transformation of dimensionality that is specific to the dataset that is being analyzed. The authors claim that this approach combines computational efficiency with higher resolution than that achievable by methods that use reduced k-mer spectra, that is, subsets usually called ”sketches” or ”signatures”. The manuscript is generally written well. The flow charts help a lot. However, the method is not easy to understand by biologists and, I assume, even by some bioinformaticians. I list some questions below so the authors can improve the explanation of the method. As for the efficiency of the method, the one concern I have is that there is no comparison to other methods. The authors make the reasonable claim that the resolution of KPop is higher than that of Mash because Mash uses reduced k-mer spectra but there are no data to support that claim. Also, the authors do not seem to be aware of the sourmash method, https://sourmash.readthedocs.io/en/latest/ , which is similar to Mash, and can also be used on unassembled sequences. The sourmash tool can use different scaling factors from 1 (no reduction) to 1000 or higher (using only 1 in a thousand k-mer of the k-mer spectrum). Therefore, my main comment is that the authors should not make a claim that their results are better than those of other methods, like Mash and sourmash (although not specifically mentioned), without showing results from a comparison of these methods. The authors say that a direct comparison with Mash is not possible (page 13, line 31-34) but accuracy of classification using Mash or sourmash with KPop should be possible.

We have now added extensive comparisons and partially rewritten the manuscript in order to reflect them. One of the key messages of our paper, and a reason why we had not initially added comparisons, is that our KPop method is able to directly provide an embedding of sequences into a low-dimensional space, while the methods mentioned by the Reviewer only allow for the computation of genomic distances between sequences. I.e., KPop is able to transform sequences into vectors in a low-dimensional space, from which distances can then be computed if needed, while other methods are only able to compute distances. That is an essential feature as it allows us to train classifiers directly from vectors, which is something that other methods cannot do and makes direct comparisons difficult. That is also the main reason why we had originally refrained from comparing KPop with other methods. We have now clarified these concepts in the new Figure 8. We have also added several comparisons with minimiser-based methods (mash, Sourmash) done as explained in the last section of the Methods, i.e., using a K-NN classifier that can be trained from distances alone. Note that we were reluctant to be too critical of existing methods such as mash or Sourmash, because, despite being currently mis-used out of their applicability range, they were never explicitly presented by their authors as able to address the type of fine-scale classification problems that we solve here.

Also, what is the actual resolution of KPop? If you had two outbreaks of Escherichia coli or Listeria monocytogenes, could KPop distinguish them from each other? I suggest to use the datasets by Timme et al

https://www.ncbi.nlm.nih.gov/pmc/articles/PMC5782805/ and report if KPop can correctly assign a query genome to different outbreaks. At the end, I think, that is one of the main applications envisioned for KPop.

We show that KPop has a higher resolution than existing tools, for example to distinguish between the different lineages of M. tuberculosis or the variants of SARS-CoV-2 which have a common ancestors only a few years ago. Whether it can assign genomes to different outbreaks depends on exactly what is meant by ‘outbreak’ and therefore we prefer to talk about lineages. For example in the simulated dataset of SARS-CoV-2 genomes we show that we are able to separate lineages that are separated by only a few years of evolution. We have also added a new application to the dataset of Blackwell et al., 2021 (doi.org/10.1371/journal.pbio.3001421) showing that we can distinguish between species, including closely related Enterobacterales such as E. coli and L. monocytogenes.

Some more specific questions/comments follow:

• In the abstract, the only indication of the high resolution of the method is the word ”lineages” implying that KPop can be used to distinguish genomes of closely related strains belonging to the same species. I suggest to point out the advantage of KPop to distinguish closely related strains more specifically.

We thank the Reviewer for this remark. We have edited the abstract to stress the high resolution of our method.

• In the general overview of the workflow, the authors say ”KPop can be applied to samples with an arbitrary k-mer composition. What is meant with ”arbitrary” here? What would be a ”non-arbitrary” k-mer composition? Also, none of the other k-mer-based methods I am aware of requires read alignment and several other methods do not require assembly. Therefore, this is not something specific to KPop but it is a general advantage of k-mer-based methods. Therfore, this should be clarified.

We have now removed this sentence which was open to misinterpretation, as correctly pointed out by the Reviewer. In fact, we substantially rewrote the introduction in order to make it clearer and shorter, and we think this work has significantly improved the readability of the manuscript.

• Same as above, k-mer based methods in general are not limited to core genes and generally use all (or a representation of all) genes in a genome.

We have rephrased to clarify that this was a comparison with alignment-based methods, not with other methods based on k-mers.

• Figure 2B is not intuitive to understand. The authors should explain the location of the dots on the plot and why that shows that the method worked. Also, the authors talk about a ”closest class (page 6, line 48) but no description of what breadth these classes have is provided. In lines 60-62, the authors talk about ”closely related”. That is a vague term. I suggest to report how many SNPs distinguished classes and how many SNPs distinguished these lineages. This goes back to my overall question of resolution: how different do two classes need to be for KPop to correctly assign genomes to them?

We have added an explanation of how this Figure works in the main text.

• In Figure 6, the color scheme should be explained better. I guess that red represents the higher confidence but that is not clear from the legend.

We have clarified this point in the legend of Figure 6.

• Choice of k-mer. The authors point out on page 14, lines 51-56, that the choice of k needs to be empirically chosen and be the ”smallest value for which the results become stable”. I guess that many readers have no idea what that means and would get stuck here before even starting to use KPop. Do the authors provide a detailed documentation with an example dataset to show to users how to choose k for their dataset? Also, it would be useful to report in the various result sections which k was used. That would help users choose k when they want to use Kpop with similar datasets.

We have now re-written and expanded our explanation on the choice of k in the Methods (section KPopCount – Choice of k).

• Finally, could the author describe, or give an example, of how the actual results look like? For example, having a set of classes and a set of query genomes and running KPop, in what format is the user given the results to which class each query genome belongs? I guess it is a table but it would be useful to have a short paragraph that describes what the output files look like and how to read them as part of the manuscript.

We have now added: (1) a quick start guide to the GitHub repository and to the documentation; (2) a new Figure 8, which details the overall KPop strategy and data formats) (3) a new Figure 9, which details the main possible workflows that can be performed with each command (4) more information in the Methods. Hopefully this additional material will help clarify most of the questions raised by the Reviewer.

Reviewer #3

Didelot and Ribeca present KPop, a k-mer based method for assembly free genome comparisons. The innovation in this approach seems to be a transformation of the k-mer profiles termed ”twister” which allows for more efficient comparisons by excluding unimportant k-mers.

Major comments:

• The manuscript is written in a style that is often used in computer science conference submissions, which might impede readability for the average Genome Biology reader and in it’s current form, the manuscript fails to convince readers that this is not just ”another k-mer method”.

We have edited the manuscript to make it less technical and to add explanations where needed. We have also added several comparisons with existing methods, so that it’s hopefully much clearer now what the method can do.

• One of the biggest shortcomings of the manuscript is the complete lack of comparative benchmarking. It is undenyable that other tools exist that can perform the analyses and task that KPop is advertised to do, so the question is: does this tool excel in certain tasks and is it better than other tools. Such benchmarks should include other tools the authors mentioned but also specific adaptations such as sourmash and kmer based tools with specific purpose such as ska + kSNP and Kraken.

We have now added extensive comparisons and partially rewritten the manuscript in order to reflect them. One of the key messages of our paper, and a reason why we had not initially added comparisons, is that our KPop method is able to directly provide an embedding of sequences into a low-dimensional space, while the methods mentioned by the Reviewer only allow for the computation of genomic distances between sequences. I.e., KPop is able to transform sequences into vectors in a low-dimensional space, from which distances can then be computed if needed, while other methods are only able to compute distances. That is an essential feature as it allows us to train classifiers directly from vectors, which is something that other methods cannot do and makes direct comparisons difficult. That is also the main reason why we had originally refrained from comparing KPop with other methods. We have now clarified these concepts in the new Figure 8. We have also added several comparisons with minimiser-based methods (mash, Sourmash) done as explained in the last section of the Methods, i.e., using a K-NN classifier that can be trained from distances alone. Note that we were reluctant to be too critical of existing methods such as mash or Sourmash, because, despite being currently mis-used out of their applicability range, they were never explicitly presented by their authors as able to address the type of fine-scale classification problems that we solve here.

• How does KPop perform at larger phylogenetic / taxonomic distances? Genus, Family, etc What happens when the correct ”class” is missing? (Also, using ”class” in anything about taxonomic assignment is obviously not optimal as Class is also a taxonomic rank)

Classification at a higher taxonomic level becomes easier, which is why we focus on finer inference. To make this point more clearly, we have added a new section in which we show KPop has perfect results on classification of data from Blackwell et al., 2021 (doi.org/10.1371/journal.pbio.3001421) into 20 species. Classifiers by definition always assign test cases to one of the classes which were present in the training set. If a class is not present in the training set then it is obviously impossible to classify an object according to it. However, our method might be able to hint to the fact that the sequence belongs to a new class, and that a new class exists, by returning a large distance from the representatives of all classes. We are unable to use another word than class even though there is a taxonomic rank called Class, because this is the terminology always used in classification problems. We have edited the manuscript to avoid using the word class when it was not necessary.

• The results are a mix of methods and results and discussion. Since this is a methods paper, some methods have to be in the results but the authors should carefully check the manuscript to make distinctions clear.

We have edited the manuscript to try and make this distinction clearer.

Minor comments:

• Regarding the existing evaulation of the method: What is the value of reporting that the second closest class is the correct one? Please explain this within the manuscript

We have added an explanation in the manuscript.

• Page 9, Line 42-49: Without showing some examples and investigating them in-depth this statement should be removed. Annotations are always an issue, but this seems very handwavy.

We have rephrased this statement to make it less assertive: we only mean to say that it is a possibility but we cannot be sure of it.

• Page 9, Line 50: Could the authors share why sequencing technology or coverage (above a certain minimal threshold that the authors also implement in some form) would influence their or any similar method?

We have rephrased this sentence.

• Page 10, Line 60: It would be good if the authors could explain this significantly worse accuracy for SARS-CoV-2

An explanation is provided on page 11.

• Figures: There has to be a better visualization of the results. Fig.2bc, Fig5b and Fig6 are all quite unituitive.

We have added in the main text an explanation for how Figures 2B, 2C and 5B were constructed. We have expanded the legend of Figure 6 to clarify the meaning of the colors.

**2^nd^ round**

**Reviewer 1**

Review: KPop: Accurate, assembly-free, and scalable comparative analysis of microbial genomes

I thank the authors for their efforts in revising the manuscript. They have addressed most of my concerns but there are a few remaining issues that need to be dealt with prior to publication.

Major issues:

- The pdf containing the ‘differences’ with the original text does not match up with the revised submission. I have assumed that the incorrect PDF was submitted and I have concentrated on the updated review contained in the differences file by referring to sentences rather than line numbers. This must be addressed prior to publication.

- The simulated SARS-CoV-2 results are likely to be substantially simpler due to the larger genetic distances between lineages than observed in the large global dataset. It seems likely that the increased error rate observed in the global dataset is driven by very closely related lineages. Thus, I disagree with the authors that the errors might be:

“due to the fact that the sequences present in the dataset [54] for founding lineages, albeit being accurate at the time of their first designation, were not always updated to a more accurate classification as novel sub-lineage definitions became available.”

The authors have not compared with the state of the art Usher tool as requested. While their results have been compared to Pangolin designations, these are built using the full dataset and not the training/test combinations used in the KPop results. Ideally, Usher would be run using the same training and test sets to allow for a comparison of the accuracy of the two methods. This would help to ascertain whether the drop in accuracy between the simulated and real data is driven by the different distances between the lineages in the two datasets or problems with the designations as claimed by the authors.

- The final paragraph in the discussion suggests that KPop can be used to build high resolution phylogenies. This is not supported by the results which do not compare to any phylogenetic algorithms including those that account for recombination such as ClonalFrameML. Instead, the manuscript describes KPop’s application to clustering and classification. Either additional comparisons are needed or this paragraph should be reworded.

Minor Issues:

- “...whereas the use of minimizers is an arbitrary choice that does not guarantee optimality” - there is not a clear definition of what ‘optimal’ is in this problem and thus it is not possible to claim KPop is optimal. Consequently, this statement should be removed.

- “...Radically different from category (3), our method also produces a representation of each sample as a vector embedded in a reduced-dimension space” - Depending on the dimensions of the input data, It could be argued that a pairwise distance matrix is a reduced dimensional representation of the data. Indeed, if one were to perform Multidimensional Scaling (MDS) on such a matrix it would give a vector representation similar to that of KPop. Therefore, I don’t think this statement is justified and should be reworded.

- “...classifiers based on KPop are largely insensitive to recombination and other mechanisms that would be challenging for the models of sequence evolution underlying the construction of phylogenetic trees.”: While I appreciate the additional simulations included by the authors to consider recombination, these results are not sufficient to claim that KPop is better able to handle recombination than phylogenetic models of sequence evolution. In general, phylogenetic models often cluster genomes correctly even in the presence of recombination. Instead, it is the branch lengths that are most affected. To make this claim, the phylogeny (and branch lengths) built using KPop distances would need to be compared to a classic approach such as Gubbins or ClonalFrameML. Either these additional comparisons should be included or this statement should be removed.

- “However, we also applied sourmash [30] on the same dataset which also classified this genome in the same way as KPop, strongly suggesting that this is a mistake in the database.” - This could also be explained by the fact that this particular classification was challenging for both algorithms. This seems reasonable given they both rely on k-mers.

- “This contrasts with a disquieting trend…” I think this statement is overly inflammatory. The vast majority of fine scale bacterial genomics currently relies on building phylogenies or fine-scale SNP based analyses. Scalability is typically addressed by first clustering genomes into more closely related subsets. Mash and sourmash are not typically used at the fine-scale and thus I think this statement should be removed or weakened.

- “KPop is the only k-mer based method we are aware of that is capable of explicitly embedding k-mer spectra in a reduced-dimensionality space” - I disagree with this statement. As stated previously, it could be argued that a distance matrix is a reduced dimensional representation. Moreover, although I agree with the authors that minhash based representations are not as easily used as input to machine learning classifiers they are still reduced dimensional representations and thus this statement is false.

- I believe that the authors have compared adequately with minhash-like algorithms. Therefore, I think the statement that “As explained in the last section of the Methods, this rules out a direct comparison with…” should be removed. It is rare that two approaches are directly comparable in terms of methodology. However, KPop is solving a problem that Mash and sourmash are routinely used to solve.

**Reviewer 2**

The authors answered all my comments to my satisfaction. The manuscript improved considerably.

**Reviewer 3**

Didelot and Ribeca present a revised version of their manuscript describing a k-mer based method for assembly free genome comparisons.

The authors have now added comparisons to mash and sourmash. Sadly they don't describe well how these tools were used. Hence it remains hard to assess if e.g. the difference in methods or the difference in selected k-mer is more impactful (this is just one example, the accuracy estimation as a whole remains obscure). Further, I don't understand why the authors don't use intrinsic classification methods (implemented e.g. in sourmash) when they compare classification accuracy and instead use some kind of home brew to adapt the results of other methods. The authors mention that these tools are currently being misused, but I have the impression that it's mainly the authors mis-using other tools which leads to sub-par results for mash and sourmash. Obviously, this is just an impression as the usage of sourmash and mash is not described in sufficient detail to be replicated.

It's also unclear why the authors didn't compare to other methods, in particular methods that were designed with the very tasks that are compared here. I listed some previously, but the task of finding and comparing to the state of the art falls on the authors. A somehow defined "most popular method" is not the one method that should be compared to. To a critical eye, the presented comparison implies cherry picking.

The authors mention that other methods are not comparable as none output the k-mer profiles in an embedding space. These notions should be removed. The authors can highlight the benefit of having such embeddings but they have to find ways to compare results to existing tools not the other way around.

Previously I commented:

How does KPop perform at larger phylogenetic / taxonomic distances? Genus, Family, etc

What happens when the correct ”class” is missing?

This question still stands and is unanswered within the manuscript. The authors rebuttal is inconcrete and in part wrong (higher taxonomic levels can prove difficult for k-mer based methods when closely related sequences are missing from the database). Further, classification obviously can also leave input unclassified when none of the existing classes fit. The second question remains relevant and needs to be addressed within the manuscript

Previously, I also commented:

Regarding the existing evaluation of the method: What is the value of reporting that the second closest class is the correct one? Please explain this within the manuscript

The authors answer that this has been added, but I cannot find it. The value of reporting that the second closest class is the correct one remains unclear!

Further, the figures 2bc, 5b and 6 are still obscure, though better described now. Their purpose remains unknown and they are unreadable (points too small + overlapping). A different visualization is necessary to convey the information indended to be shown.

**Authors’ response**

We thank the Reviewers for their thorough revision of the manuscript.We have addressed all the points raised as detailed below. We would like to note that some of the responses required a very signi_cant amount of e_ort and time on our part { for instance, some of the comparisons that were requested for the SARS-CoV-2 example alone took several weeks of computing on a large HPC node. In addition, we have added plenty of information to the Methods and, in particular, the KPop GitHub repository, so as to help new users to better grasp how the method works and how to use it in the best possible way. We hope Editor and Reviewers will take this into account when they re-evaluate the manuscript.

Reviewer #1

I thank the authors for their e_orts in revising the manuscript. They have addressed most of my concerns but there are a few remaining issues that need to be dealt with prior to publication. Major issues

_ The pdf containing the \di_erences" with the original text does not match up with the revised submission. I have assumed that the incorrect PDF was submitted and I have concentrated on the updated review contained in the di_erences _le by referring to sentences rather than line numbers. This must be addressed prior to publication.

We are surprised by this and apologise if there was a discrepancy between the clean _le and the one highlighting the di_erences. It is di_cult to keep track of changes when using latex. However, we have made sure that for this second round of revision the di_erence _le shows exactly the edits made between the version submitted in our _rst revision and the latest manuscript submitted in this revision.

_ The simulated SARS-CoV-2 results are likely to be substantially simpler due to the larger genetic distances between lineages than observed in the large global dataset. It seems likely that the increased error rate observed in the global dataset is driven by very closely related lineages. Thus, I disagree with the authors that the errors might be: \due to the fact that the sequences present in the dataset [54] for founding lineages, albeit being accurate at the time of their _rst designation, were not always updated to a more accurate classi_cation as novel sub-lineage de_nitions became available."

We have removed this sentence which suggested that the Pangolin classi_cations might be incorrect. Instead, we have added a full comparison of the classi_cation of KPop against Usher as suggested by the reviewer and described below. We note that most instances of misclassi_cation are due to heterogeneities between classes, and illustrate this with an example in the new Figure S4.

_ The authors have not compared with the state of the art Usher tool as requested. While their results have been compared to Pangolin designations, these are built using the full dataset and not the training/test combinations used in the KPop results. Ideally, Usher would be run using the same training and test sets to allow for a comparison of the accuracy of the two methods. This would help to ascertain whether the drop in accuracy between the simulated and real data is driven by the di_erent distances between the lineages in the two datasets or problems with the designations as claimed by the authors.

We followed the reviewer's suggestion of running Usher on exactly the same training and testing datasets. We found that Usher correctly classi_ed 97.5% of the sequences, if we consider that the Pangolin classi_cations are the ground truth. To demonstrate the exibility of our approach we also added a simple random forest-based KPop classi_er, which achieves a better accuracy (98.1%). However, it should be noted that running Usher on this dataset required in excess of 100 times more computational resources than running KPop | equivalent to roughly two weeks of computation on a relatively large HPC node using 64 CPUs | and also required as input a phylogeny of the training set with 600,000 leaves, which would require a similar amount of time to be generated as the one taken by Usher. We have added a paragraph in the manuscript to describe this comparison between KPop and Usher on this dataset.

_ The _nal paragraph in the discussion suggests that KPop can be used to build high resolution phylogenies. This is not supported by the results which do not compare to any phylogenetic algorithms including those that account for recombination such as ClonalFrameML. Instead, the manuscript describes KPop's application to clustering and classi_cation. Either additional comparisons are needed or this paragraph should be reworded.

Yes, this _nal paragraph of the discussion was only making a suggestion for future work, rather than reporting on something we claim to have done. We intend to pursue this line of work in the future. We have edited this paragraph to make this completely clear.

Minor Issues

_ \...whereas the use of minimizers is an arbitrary choice that does not guarantee optimality" - there is not a clear de_nition of what `optimal' is in this problem and thus it is not possible to claim KPop is optimal. Consequently, this statement should be removed.

We have edited this text to remove this statement on optimality.

_ \...Radically di_erent from category (3), our method also produces a representation of each sample as a vector embedded in a reduced-dimension space" - Depending on the dimensions of the input data, It could be argued that a pairwise distance matrix is a reduced dimensional representation of the data. Indeed, if one were to perform Multidimensional Scaling (MDS) on such a matrix it would give a vector representation similar to that of KPop. Therefore, I don't think this statement is justi_ed and should be reworded.

We have reworded this sentence as suggested by the reviewer.

_ \...classi_ers based on KPop are largely insensitive to recombination and other mechanisms that would be challenging for the models of sequence evolution underlying the construction of phylogenetic trees.":

While I appreciate the additional simulations included by the authors to consider recombination, these results are not su_cient to claim that KPop is better able to handle recombination than phylogenetic models of sequence evolution. In general, phylogenetic models often cluster genomes correctly even in the presence of recombination. Instead, it is the branch lengths that are most a_ected. To make this claim, the phylogeny (and branch lengths) built using KPop distances would need to be compared to a classic approach such as Gubbins or ClonalFrameML. Either these additional comparisons should be included or this statement should be removed.

We have rephrased this text to make it clear that we are not claiming to have compared to phylogenetic methods.

_ \However, we also applied sourmash [30] on the same dataset which also classi_ed this genome in the same way as KPop, strongly suggesting that this is a mistake in the database." - This could also be explained by the fact that this particular classi_cation was challenging for both algorithms. This seems reasonable given they both rely on k-mers.

Thanks, we have added a sentence to make this point.

_ \This contrasts with a disquieting trend..." I think this statement is overly inammatory. The vast majority of _ne scale bacterial genomics currently relies on building phylogenies or _ne-scale SNP based analyses. Scalability is typically addressed by _rst clustering genomes into more closely related subsets. Mash and sourmash are not typically used at the _ne-scale and thus I think this statement should be removed or weakened.

We have removed this statement.

_ \KPop is the only k-mer based method we are aware of that is capable of explicitly embedding k-mer spectra in a reduced-dimensionality space" - I disagree with this statement. As stated previously, it could be argued that a distance matrix is a reduced dimensional representation. Moreover, although I agree with the authors that minhash based representations are not as easily used as input to machine learning classi_ers they are still reduced dimensional representations and thus this statement is false.

We have edited this sentence to clarify our meaning, which is that KPop is natively able to produce an embedding in reduced-dimension space, rather than having to be generate by postprocessing the distances.

_ I believe that the authors have compared adequately with minhash-like algorithms. Therefore, I think the statement that \As explained in the last section of the Methods, this rules out a direct comparison with..." should be removed. It is rare that two approaches are directly comparable in terms of methodology. However, KPop is solving a problem that Mash and sourmash are routinely used to solve.

We have removed this statement.

Reviewer #2

The authors answered all my comments to my satisfaction. The manuscript improved considerably.

Thank you!

Reviewer #3

Didelot and Ribeca present a revised version of their manuscript describing a k-mer based method for assembly free genome comparisons.

_ The authors have now added comparisons to mash and sourmash. Sadly they don't describe well how these tools were used. Hence it remains hard to assess if e.g. the di_erence in methods or the di_erence in selected k-mer is more impactful (this is just one example, the accuracy estimation as a whole remains obscure). Further, I don't understand why the authors don't use intrinsic classi_cation methods (implemented e.g. in sourmash) when they compare classi_cation accuracy and instead use some kind of home brew to adapt the results of other methods. The authors mention that these tools are currently being misused, but I have the impression that it's mainly the authors mis-using other tools which leads to sub-par results for mash and sourmash. Obviously, this is just an impression as the usage of sourmash and mash is not described in su_cient detail to be replicated. It's also unclear why the authors didn't compare to other methods, in particular methods that were designed with the very tasks that are compared here. I listed some previously, but the task of _nding and comparing to the state of the art falls on the authors. A somehow de_ned \most popular method" is not the one method that should be compared to. To a critical eye, the presented comparison implies cherry picking.

We believe there is a basic misunderstanding between us and the Reviewer on this point. When we talk about \classi_cation" in our manuscript, we are not actually talking about taxonomic classi_cation, but about classi_cation in the usual statistical sense, as used for instance in machine learning and related disciplines. There one has a set of sequences whose mutual relation is unspeci_ed; and, given some new test sequence, one just wants to _nd which ones of the known sequences the new sequence is more similar to, instead of getting a taxonomic classi_cation for the test sequence. For instance, in the case of a new SARS-CoV-2 sequence one might be just interested in _nding what its \type" is rather than in determining the full placement of the new sequence within the full SARS-CoV-2 sequence tree | in a sense, one would just like to understand which \leaf" of a phylogenetic tree the test sequence is closest to. This kind of \partial" classi_cation problem is common in public-health settings, and the applications showed in the paper had focused on it. That is why we had not originally benchmarked against Sourmash built-in classi_cation method, which is a taxonomic one. We have now added exact details of how mash and Sourmash were used previously. We have also added an attempt at classi_cation using the Sourmash built-in classi_er for the dataset with 20 bacterial species. Finally, we have added a full taxonomic classi_cation using both Sourmash and KPop for a new application based on the TARA Oceans metagenome project, which is the dataset used in the Sourmash user guide to demonstrate the use of the Sourmash built-in taxonomic classi_er.

_ The authors mention that other methods are not comparable as none output the k-mer pro_les in an embedding space. These notions should be removed. The authors can highlight the bene_t of having such embeddings but they have to _nd ways to compare results to existing tools not the other way around.

We have removed this statement.

_ Previously I commented: How does KPop perform at larger phylogenetic / taxonomic distances? Genus, Family, etc What happens when the correct \class" is missing?

This question still stands and is unanswered within the manuscript. The authors rebuttal is inconcrete and in part wrong (higher taxonomic levels can prove di_cult for k-mer based methods when closely related sequences are missing from the database). Further, classi_cation obviously can also leave input unclassi_ed when none of the existing classes _t. The second question remains relevant and needs to be addressed within the manuscript

We have added a completely new KPop-based workow to perform taxonomic classi_cation at di_erent levels, which illustrates well how classi_cation becomes increasingly di_cult as _ner taxonomic units are being used. When the correct \class" is missing, for example a species present in the test dataset that was not found in the training dataset, the KPop classi_er returns the closest match at the lowest possible taxonomic level. We found in this application that KPop outperforms Sourmash at all taxonomic classi_cation levels.

_ Previously, I also commented: Regarding the existing evaluation of the method: What is the value of reporting that the second closest class is the correct one? Please explain this within the manuscript The authors answer that this has been added, but I cannot _nd it. The value of reporting that the second closest class is the correct one remains unclear!

The following text was added in the previous round of revision which clari_es our reason for reporting the distance to the second closest class: \We computed the distance of every test genome to each of the classes. For each genome the class that had the lowest distance was the correct one, which means that all genomes were correctly classi_ed. A measure of classi_cation certainty can be obtained by comparing the distance from each genome to the classes with the lowest and second lowest distance. Figure 3B shows each test genome as a dot, with the x-axis representing the distance to the class with the lowest distance, and the y-axis representing the distance to the class with the second-lowest distance."

_ Further, the _gures 2bc, 5b and 6 are still obscure, though better described now. Their purpose remains unknown and they are unreadable (points too small + overlapping). A di_erent visualization is necessary to convey the information indended to be shown.

These _gures all follow the same format, showing on the x-axis the distance to the best class and on the y-axis the distance to the second class. The reason for showing this has been clari_ed, as detailed in our response to the previous comment above. It is indeed not possible to see exactly all points, since there is a point for each of the 10,000 genomes for example in Figure 5B. However, seeing each point separately is not needed to reach our conclusions about the performance of the classi_er.

**3^rd^ round**

**Reviewer 1**

|  | I thank the authors for their efforts in revising the manuscript. They have addressed my major concerns, although I found the inability of some hash based algorithms to classify accurately at the species level surprising. I have a few remaining minor points.   - Figure 5 and 6 need larger font sizes  - It’s possible I’ve missed them, but the supplementary figures did not appear to have captions. These should be added.  - p21 - line 50: I could not find “Correspondence Analysis” mentioned in ref 68. |
| --- | --- |

**Reviewer 3**

Didelot and Ribeca present a second revision of their manuscript on their tool KPop.
The authors have added more analyses as well as additional information. I welcome that this informatins has been added now, especially in the Methods section as it makes it easier to reproduce and to review the manuscript. While I value the additional work of the authors, I do find the initial statement of the rebuttal slightly odd. The peer review process is supposed to ensure a certain and improve the quality of the manuscript. Hence, it would be nice if the authors would appreciate the reviewers work and participate in the peer review process in a more pro-active manner.

Regarding the authors to my point 1, in the last revision:
I am not sure where the authors see a "basic misunderstanding". I think we all agree that classification is the assignment to a "class", group or cluster. Further, I am not sure why the authors imply that I focus on "taxonomic classification" as I don't mention the term or allude to it.
The question remains why the authors use use a non-benchmarked, home brewed method for classification of the sourmash results.
sourmash actually provides a method that can assign sequences to lineages, clusters or classes. Given this is within the "sourmash tax" functionality (option -lins), but provides exactly what the authors want to perform (it is not limited to taxonomic classification).
Also, I never asked for a "full placement" into a tree as the authors insinuate. All I am asking is for them to correctly use the tools they compare to and report their methodology. As I mentioned previously, other tools are dedicated to provide the functionality the authors want to compare to but (again) the authors have not done their due diligence to find and compare to these tools (apart from the new comparison to the usher tool (as suggested by another reviewer)). At this stage, the authors should consider comparing their results to those of sylph (https://doi.org/10.1101/2023.11.20.567879), kSNP (https://doi.org/10.1093/bioinformatics/btv271) and SKA (https://doi.org/10.1101/453142). kSNP and SKA (which I already mentioned during my first round of comments) use split kmers and sylph uses a novel approach to kmer based (meta)genomics analyses. kPop is presented as being good in many different applications, but needs to be compared to tools that excel in these specific areas.

Again, I thank the authors for adding to the methodology section for sourmash. Yet, I am confused by the authors use of sourmash lca. When I went onto the sourmash website to understand what sourmash lca does, I was greeted by following message: "Attention. We no longer recommend using sourmash lca for taxonomic analysis; please use sourmash tax instead. See taxonomic profiling with sourmash for more information." A quick check on the github repo of sourmash website revealed that this message has been there since approx. 6 months, hence I think it is the authors due diligence to adhere to this recommendation. Especially as their results for sourmash mirror the reasons given on the sourmash website why sourmash tax is recommended over sourmash lca.
During the last revision, I expected that one the reasons for the underperformance of sourmash would be the choice of a k-mer (which obviously has to be sensible for different scenarios), but I obviously could not make this claim as the methodology was not clearly described. The authors chose the default k=31 which is most probably sub-optimal for the tasks at hand. This should be at least mentioned in the discussion.

Figure S1, S2 and the relevant paragraph on page 8. When I look at the supplementary figures, I see one figure indicating that sourmash does not enough resolution (with very little noise) and then one that has high resolution but a lot of noise, i.e. 2 different types of errors. Which leads me to believe that sourmash could have been applied wrongly here.
It seems that the authors did not follow the instructions for providing a sensible k as described on the sourmash website and k = 31 as described in the methods might not be a sensible choice here. The authors describe their own procedure to optimize k for KPop (yet I couldn't find which k was actually used for which analyses). The least the authors can do is run tool comparisons using the same k for KPop and the other tool (as well as the default and in the best case a sensible k). The question "is it the k or is it the tool?" remains relevant.

Page 8: "A measure of classification certainty can be obtained by comparing the distance from each genome to the classes with the lowest and second lowest distance."
While I appreciate that the authors have added some explanation now (after I asked for an explanation twice), yet I have to say this statement is false. The distance between the lowest and second lowest distance in the described scenario is a property of the database that is mapped against. As I mentioned before the accompanying figures (3bc and 5, 6) are borderline unreadable and the added value for readers is very low (I can and could always read them btw, but that doesn't make this figure type more useful). If the authors want to provide something useful they could (for example) show the correlation between actual distance (as simulated) and that generated by KPop against different classes.
At the very least, please mark the red points in figure 5 in the accompaying tree to give them at least a bit of meaning. Also I find it a bit off that in Figure 6, points marked in red indicate high confidence while red points in figure 5 are errors.

Figure S4 and the relevant paragraph on page 13. The figure is not legible. Please add a legend describing the colors. I would actually add more colors to mark the different clade. Currently, I cannot see if the authors statements in the paragraph are supported by the figure or not

Page 18/19: I mentioned this already, but to make sure this doesn't get lost within my previous comment: The authors spent a whole page on how to select k, but don't report which k they chose for any of the analyses. This should be an easy fix.

Comment to reviewer: "When the correct class" is missing, for example a species present in the test dataset that was not found in the training dataset, the KPop classifier returns the closest match at the lowest possible taxonomic level."
Thank you for clarifying. I would appreciate if this description would also be reflected in the manuscript. Seems an important consideration for readers.

**Authors’ response**

We thank the Reviewers for their thorough review of the manuscript. We have addressed all the points raised as detailed below, and, additionally, performed a systematic revision of the text in order to make it consistent again after all changes made during this and previous revisions. We hope the Editor and Reviewers will now _nd our manuscript to be acceptable for publication.

Reviewer #1

I thank the authors for their e_orts in revising the manuscript. They have addressed my major concerns, although I found the inability of some hash based algorithms to classify accurately at the species level surprising. I have a few remaining minor points.

_ Figure 5 and 6 need larger font sizes

That has been done now.

_ It's possible I've missed them, but the supplementary _gures did not appear to have captions. These should be added.

The legends of the supplementary _gures have been added and can be found on the last page.

_ p21 - line 50: I could not _nd 'Correspondence Analysis' mentioned in ref 68.

This reference was meant to explain what is meant by 'compositional analysis'. We have now replaced it with a better one where the links between compositional and correspondence analysis are discussed.

Reviewer #3

_ Regarding the authors to my point 1, in the last revision: I am not sure where the authors see a "basic misunderstanding". I think we all agree that classi_cation is the assignment to a "class", group or cluster. Further, I am not sure why the authors imply that I focus on "taxonomic classi_cation" as I don't mention the term or allude to it. The question remains why the authors use use a non-benchmarked, home brewed method for classi_cation of the sourmash results. sourmash actually provides a method that can assign sequences to lineages, clusters or classes. Given this is within the "sourmash tax" functionality (option -lins), but provides exactly what the authors want to perform (it is not limited to taxonomic classi_cation). Also, I never asked for a "full placement" into a tree as the authors insinuate. All I am asking is for them to correctly use the tools they compare to and report their methodology. As I mentioned previously, other tools are dedicated to provide the functionality the authors want to compare to but (again) the authors have not done their due diligence to _nd and compare to these tools (apart from the new comparison to the usher tool (as suggested by another reviewer)). At this stage, the authors should consider comparing their results to those of sylph

(https://doi.org/10.1101/2023.11.20.567879), kSNP (https://doi.org/10.1093/bioinformatics/btv271)

and SKA (https://doi.org/10.1101/453142). kSNP and SKA (which I already mentioned during my _rst round of comments) use split kmers and sylph uses a novel approach to kmer based (meta)genomics analyses. kPop is presented as being good in many di_erent applications, but needs to be compared to tools that excel in these speci_c areas.

We are glad to see that we are on the same page about this point. Certainly our intention was not to "insinuate" anything, just to (respectfully) clarify what we understood about the reviewer's comments and what we didn't. It is very possible that we misunderstood some of the reviewer's remarks, in which case we are glad to apologise. According to the reviewer's suggestion, for this revision we are not using any home-brewed method or the old sourmash lca workow, but rather following the sourmash tax workow that recently replaced t. We re-run all the relevant examples and reviewed the manuscript accordingly. Hopefully this, which required a large amount of e_ort and computation, will convince the reviewer of our goodwill. In general, this paper is not about showing that KPop is superior to each and every existing k-mer based tool, nor are we making any claims in that direction. We just want to show that KPop performs well in a number of scenarios. It would be impossible for us to prove or disprove that a tool is better than another in absolute terms, as for complex methods it is always possible to _nd speci_c datasets on which either tool is better. We also disagree that the methods mentioned by the author are especially relevant to this paper, where we already present a (too) large number of examples and comparisons with other tools. A specialised comparison to even more tools would be out of scope and is better left to follow-up papers focusing on speci_c tasks. We now mention more of the tools listed by the reviewer in the Discussion.

_ Again, I thank the authors for adding to the methodology section for sourmash. Yet, I am confused by the authors use of sourmash lca. When I went onto the sourmash website to understand what sourmash lca does, I was greeted by following message: "Attention. We no longer recommend using sourmash lca for taxonomic analysis; please use sourmash tax instead. See taxonomic pro_ling with sourmash for more information." A quick check on the github repo of sourmash website revealed that this message has been there since approx. 6 months, hence I think it is the authors due diligence to adhere to this recommendation. Especially as their results for sourmash mirror the reasons given on the sourmash website why sourmash tax is recommended over sourmash lca. During the last revision, I expected that one the reasons for the underperformance of sourmash would be the choice of a k-mer (which obviously has to be sensible for di_erent scenarios), but I obviously could not make this claim as the methodology was not clearly described. The authors chose the default k=31 which is most probably sub-optimal for the tasks at hand. This should be at least mentioned in the discussion.

The reviewer is correct that the message deprecating sourmash lca is present on the website. However, references to sourmash lca and a tutorial about its use are also still present, which confused us. For instance, the help hardcoded in our conda version of sourmash gather (version 4.8.2) still says: Use `sourmash gather` to classify a metagenome against a collection of genomes with no (or incomplete) taxonomic information. Use `sourmash lca summarize` to classify a metagenome using a collection of genomes with taxonomic information. In general, we added a comparison with sourmash at the reviewer's request, but we do not claim to be experts in its use. At any rate, we have now removed from the manuscript all the instances of sourmash lca and of our home-brewed k-NN classi_er based on sourmash distances (we still examine distances to NNs in some cases, but only to compare them to the ones generated by KPop). As per the reviewer's request we replaced such instances with sourmash tax, which yielded similar results as before (with the notable exception of the Salmonella recombination example, where sourmash tax performed as well as KPop). To address the reviewer's point about the choice of k, in addition to using the default value k = 31 we also ran all the examples with k = 21 and discussed in the text the di_erences this causes. However, the results change only marginally and our previous qualitative conclusions remain generally correct. We would like to restate that this paper is not about showing that KPop is superior to sourmash, as the two methods are not equivalent { while sourmash compares sequences by estimating k-mer based distances, KPop turns sequences into points in an abstract space. We just want to show that our method is sound and can produce meaningful results in a number of scenarios.

_ Figure S1, S2 and the relevant paragraph on page 8. When I look at the supplementary _gures, I see one _gure indicating that sourmash does not enough resolution (with very little noise) and then one that has high resolution but a lot of noise, i.e. 2 di_erent types of errors. Which leads me to believe that sourmash could have been applied wrongly here. It seems that the authors did not follow the instructions for providing a sensible k as described on the sourmash website and k = 31 as described in the methods might not be a sensible choice here. The authors describe their own procedure to optimize k for KPop (yet I couldn't _nd which k was actually used for which analyses). The least the authors can do is run tool comparisons using the same k for KPop and the other tool (as well as the default and in the best case a sensible k). The question "is it the k or is it the tool?" remains relevant.

We appreciate the reviewer's concern but do not believe the di_erence in performance to be caused by the choice of k, as the ranges and rationales for the typical values of k used by KPop and by minimiser-based methods are completely di_erent. KPop uses low values of k (by default, 12) with the idea of keeping information about the frequencies of each k-mer, while minimiser-based methods use much longer k-mer values (such as 32 or 64) with the idea of detecting presence/absence of each k-mer (it will be rare to _nd more than one 32- or 64-mer in a biological sequence, and minimiser-based methods often discard frequency information anyway). Even if that was technically possible, it would not make sense to run KPop with the same value of k as sourmash, nor run sourmash with the same value of k as KPop, as the methods are fundamentally di_erent. We have now added the values of k used for KPop to all the examples in the paper (it is most often the default value of k = 12, which should be adequate for most bacterial genomes). In the previous revision we did not try to optimise sourmash and ran it at default values. We had clearly stated this in the paper. For this revision, in order to respond to the reviewer's query, we also re-ran all the relevant examples using k = 21. In general, choosing a lower value for k improves results, but only marginally | overall, the results at k = 21 look very similar to the ones we get for the default value k = 31, in particular for the non-simulated sequencing datasets. We now mention all these considerations in the manuscript. We also added to the supplementary materials plots of the confusion matrices for sourmash tax for a number of relevant benchmarks.

_ Page 8: "A measure of classi_cation certainty can be obtained by comparing the distance from each genome to the classes with the lowest and second lowest distance." While I appreciate that the authors have added some explanation now (after I asked for an explanation twice), yet I have to say this statement is false. The distance between the lowest and second lowest distance in the described scenario is a property of the database that is mapped against. As I mentioned before the accompanying _gures (3bc and 5, 6) are borderline unreadable and the added value for readers is very low (I can and could always read them btw, but that doesn't make this _gure type more useful). If the authors want to provide something useful they could (for example) show the correlation between actual distance (as simulated) and that generated by KPop against di_erent classes. At the very least, please mark the red points in _gure 5 in the accompaying tree to give them at least a bit of meaning. Also I _nd it a bit o_ that in Figure 6, points marked in red indicate high con_dence while red points in _gure 5 are errors.

The simulation uses the phylogeny shown in Figure 5A, so there is no correct distance to each class that could be used for a correlation plot. We have added markers in the tree in Figure 5A to indicate the 11 genomes highlighted in Figure 5B. We have switched the colouring from red to cyan in Figure 5 to avoid confusion with the red colour used in Figure 6.

_ Figure S4 and the relevant paragraph on page 13. The _gure is not legible. Please add a legend describing the colours. I would actually add more colours to mark the di_erent clade. Currently, I cannot see if the authors statements in the paragraph are supported by the _gure or not

We appreciate that it might be di_cult for the reviewer to get the details of the _gure, but essentially the labels are there for illustration purposes { only the colours matter (and the _gure uses vector graphics, so it is possible to magnify it almost at will). Red represents the genomes from lineage B.1.617.2 and black the genomes from lineage AY.4 (alias of B.1.617.2.4). The legend of this Figure S4 can now be found on the last page of the manuscript, which hopefully will help clarifying.

_ Page 18/19: I mentioned this already, but to make sure this doesn't get lost within my previous comment: The authors spent a whole page on how to select k, but don't report which k they chose for any of the analyses. This should be an easy _x.

Cf above.

_ Comment to reviewer: "When the correct class" is missing, for example a species present in the test dataset that was not found in the training dataset, the KPop classi_er returns the closest match at the lowest possible taxonomic level." Thank you for clarifying. I would appreciate if this description would also be reected in the manuscript. Seems an important consideration for readers.

This sentence was added in the section \Taxonomic classi_cation":

\When the correct class is missing, for example a species present in the test dataset that was not found in the training dataset, the classi_er returns the closest match at the lowest possible taxonomic level."

**4^th^ round**

**Reviewer 3**

The authors have now performed the most essential benchmarks, and though the paper would not convince me to use the tool (not because of the quality of the tool but solely based on the written manuscript, I think the tool is actually useful), the manuscript seems to be mostly scientifically and technically sound.
I have a few minor points regarding the manuscript, but essentially would support the publication of the manuscript

I reread the introduction and on page 2, line 8 I would like the authors to add a sentence on ANI calculation tools with citations here. ANI calculation has become the de facto standard for these type of analyses.

On page 4, line 28: the authors mention: "If novel sequences that are very different from what was already present in the database become known, one just has to regenerate the twister at step (2) and re-compute reduced-dimensional signatures from the spectra, which is quick and straightforward". In the section before this statement, the authors mention earlier that this is part is "compute- and resource-intensive", please clarify.

On page 16, the authors mention" "Compared to other methods that are specifically designed to perform a single task such as establishing relations or searching for specific genetic elements, our strategy retains superior flexibility."
This is not really in line with the statements throughout the answers to the reviewer that justify the lack of a more elaborate benchmark. There are other methodologies using k-mers that have shown great flexibility and performance.

In the reviewer comments section, the authors answer: "In general, this paper is not about showing that KPop is superior to each and every existing k-mer
based tool, nor are we making any claims in that direction. We just want to show that KPop performs
well in a number of scenarios."

Please add this to the discussion. Especially as there are many advanced k-mer based methods that were not benchmarked against, so there could be a superior general strategy (a candidate would be the one underlying skani, sylph, and a few other tools by those same authors, which is also so flexible that it has lead to multiple high profile publications of different tools with different use cases but one underlying strategy). The current mention of these competing tools in the context of the whole paper needs a bit more context.

**Authors’ response**

We thank Reviewer 3 for their additional feedback. We have again addressed all the points raised as detailed below, and performed yet another thorough revision in order to improve the text of the Introduction and Conclusions wherever our formulation was suboptimal. We believe the result to be of acceptable quality and hope the Editor and Reviewers will agree and approve the manuscript for publication.

Reviewer #3

The authors have now performed the most essential benchmarks, and though the paper would not convince me to use the tool (not because of the quality of the tool but solely based on the written manuscript, I think the tool is actually useful), the manuscript seems to be mostly scientifically and technically sound. I have a few minor points regarding the manuscript, but essentially would support the publication of the manuscript.

We thank the reviewer for this assessment.

• I reread the introduction and on page 2, line 8 I would like the authors to add a sentence on ANI calculation tools with citations here. ANI calculation has become the de facto standard for these type of analyses.

This is fair enough. We now mention ANI in several sentences and have added references to a few implementations.

• On page 4, line 28: the authors mention: “If novel sequences that are very different from what was already present in the database become known, one just has to regenerate the twister at step (2) and re-compute reduced-dimensional signatures from the spectra, which is quick and straightforward”.

In the section before this statement, the authors mention earlier that this is part is “compute- and resource-intensive”, please clarify.

Agreed – during previous revisions we had modified other parts of the manuscript to emphasize that computing the twister is expensive, but we apparently overlooked this sentence. We have now eliminated its “which is quick and straightforward” part.

• On page 16, the authors mention “Compared to other methods that are specifically designed to perform a single task such as establishing relations or searching for specific genetic elements, our strategy retains superior flexibility.” This is not really in line with the statements throughout the answers to the reviewer that justify the lack of a more elaborate benchmark. There are other methodologies using k-mers that have shown great flexibility and performance.

We can see the reviewer’s point, even though it is also true that KPop is extremely flexible, as witnessed by the fact that during the review process we have been asked to benchmark it against a number of k-mer based methods. We changed the sentence to “our strategy retains great flexibility” which hopefully does not implicitly convey an idea of superiority backed by benchmarks.

• In the reviewer comments section, the authors answer: “In general, this paper is not about showing that KPop is superior to each and every existing k-mer based tool, nor are we making any claims in that direction. We just want to show that KPop performs well in a number of scenarios.” Please add this to the discussion. Especially as there are many advanced k-mer based methods that were not benchmarked against, so there could be a superior general strategy (a candidate would be the one underlying skani, sylph, and a few other tools by those same authors, which is also so flexible that it has lead to multiple high profile publications of different tools with different use cases but one underlying strategy). The current mention of these competing tools in the context of the whole paper needs a bit more context.

As requested by the reviewer, we have added a sentence to that effect at the beginning of the Discussion. We have also revised the Discussion in order to temper some statements and make them more consistent with the first paragraph. We would also like to point out that the main target of our comparison are not really k-mer based methods in general but rather minimizer-based ones, which are a more limited subset. Hopefully this is now properly conveyed by the Discussion
